# KS-Detect – Validation of Solar Thermal PCR for the Diagnosis of Kaposi’s Sarcoma Using Pseudo-Biopsy Samples

**DOI:** 10.1371/journal.pone.0147636

**Published:** 2016-01-22

**Authors:** Ryan Snodgrass, Andrea Gardner, Li Jiang, Cheng Fu, Ethel Cesarman, David Erickson

**Affiliations:** 1 Sibley School of Mechanical and Aerospace Engineering, Cornell University, Ithaca, New York, United States of America; 2 Department of Pathology and Laboratory Medicine, Weill Cornell Medical College, New York, New York, United States of America; 3 Meinig School of Biomedical Engineering, Cornell University, Ithaca, New York, United States of America; Institut Pasteur of Shanghai, Chinese Academy of Sciences, CHINA

## Abstract

Resource-limited settings present unique engineering challenges for medical diagnostics. Diagnosis is often needed for those unable to reach central healthcare systems, making portability and independence from traditional energy infrastructure essential device parameters. In 2014, our group presented a microfluidic device that performed a solar-powered variant of the polymerase chain reaction, which we called solar thermal PCR. In this work, we expand on our previous effort by presenting an integrated, portable, solar thermal PCR system targeted towards the diagnosis of Kaposi’s sarcoma. We call this system KS-Detect, and we now report the system’s performance as a diagnostic tool using pseudo-biopsy samples made from varying concentrations of human lymphoma cell lines positive for the KS herpesvirus (KSHV). KS-Detect achieved 83% sensitivity and 70% specificity at high (≥10%) KSHV+ cell concentrations when diagnosing pseudo-biopsy samples by smartphone image. Using histology, we confirm that our prepared pseudo-biopsies contain similar KSHV+ cell concentrations as human biopsies positive for KS. Through our testing of samples derived from human cell lines, we validate KS-Detect as a viable, portable KS diagnostic tool, and we identify critical engineering considerations for future solar-thermal PCR devices.

## Introduction

Kaposi’s sarcoma (KS) is one of the most common cancers in untreated individuals who are afflicted with human immunodeficiency virus (HIV)[1]. The epidemic form of KS occurs primarily in sub-Saharan Africa, accounting for almost 90% of all KS cases, and has a low 5 year survival rate at only 12%[1,2]. KS formation is a result of infection with Kaposi’s sarcoma herpesvirus (KSHV), also known as human herpesvirus 8 (HHV8), believed to be transmitted primarily through saliva[1,3,4]. Although there is currently no cure for KS, treatment does exist. Antiretroviral therapy (ART) and combination antiretroviral therapy (cART) have been shown to improve both the course of the disease and long-term survival, but largely for KS diagnosed early[5–8]. A 2010 South African study consisting of 215 epidemic KS patients reported roughly a 20% increase in one-year survival rates for patients receiving cART versus those who were not[9]. Furthermore, survival was about 30% lower for those diagnosed at later stages of the disease versus those diagnosed in earlier stages. A tool for the timely diagnosis of KS in resource-limited settings is needed and could help to decrease KS mortality.

Diagnosis of KS is difficult. KSHV detection by serological assay is impractical, as much of sub-Saharan Africa has a KSHV seroprevalence rate above 50%[1,10] (KSHV infection is necessary for KS formation, but is not sufficient). KSHV viremia is also inadequate for diagnosis because the virus can be found in the blood of patients without KS, and not all individuals with KS will have viremia[11,12]. Diagnosis by pathology following biopsy is possible, but is limited without immunohistochemistry to detect viral protein: a 2012 study found that pathology performed in Uganda and Kenya achieved only 72% sensitivity and 84% specificity for KS diagnosis when US pathology that included immunohistochemistry was taken as the gold standard[13]. A highly specific alternative to serology and pathology exists in the form of nucleic acid diagnostics. In 1994 it was discovered that DNA subfragments in the KSHV episome were amplified only in biopsy samples that were known by microscopy to be positive for KS[14].

The polymerase chain reaction (PCR) achieves amplification of specific nucleic acids by cycling DNA through three temperature zones. An example of a successful PCR-based diagnostic system is the GeneXpert, which has been used as a tuberculosis diagnostic in resource-limited settings for many years. As of September 2014, the GeneXpert system had been implemented in 110 low or middle-income countries[15], and successfully decreased the median time to treatment for tuberculosis patients from 70 to 28 days[16]. While the GeneXpert system is suitable for central healthcare institutions, its lack of portability and its reliance on electricity and supporting equipment make it inapt for use in resource-limited settings. An ideal diagnostic device for resource-limited settings should be portable, low-cost, and capable of operation independent of energy infrastructure[17], allowing the device to be taken to patients who are unable to reach secondary or central healthcare institutions.

Lab-on-a-chip (LOC) microfluidic PCR devices have been in development since the 1990s[18], and present strong opportunities to reduce the cost, size, and energy requirements of diagnostic tools. A solar thermal heated, continuous-flow, microfluidic PCR chip was first demonstrated by our group in 2014[19]. Its key advantage over other LOC, PCR devices is that the primary energy requirement for PCR (heating of the three temperature zones) is accomplished solar thermally, making it well-suited for use in locations with unreliable power sources but with ample access to sunlight. In our earlier publication, we discussed the operating principles of our solar thermal PCR microfluidic chip, tested it using plasmid DNA samples, and explored how some experimental conditions (e.g. time of day or ambient temperature) affected our ability to perform PCR using sunlight. However, we had not characterized its performance (i.e. sensitivity and specificity) with samples derived from human cell lines (important because real biopsy samples will contain similar PCR inhibitors), nor had we presented a device suitable for deployment.

We now present the KS-Detect: an integrated solar thermal PCR system suitable for field deployment in resource-limited settings and targeted towards the diagnosis of Kaposi’s sarcoma. In this paper, we introduce the KS-Detect system, describe how to operate it, and validate its ability as a diagnostic tool using DNA from pseudo-biopsy samples extracted using a simplified methodology and not requiring complex laboratory equipment. We show that the KS-Detect successfully amplifies samples with KSHV+ concentrations similar to those of real human biopsy samples positive for KS, report sensitivity and specificity results, and comment on critical engineering considerations for future solar thermal PCR systems.

### The KS-Detect system

**Fig 1:**
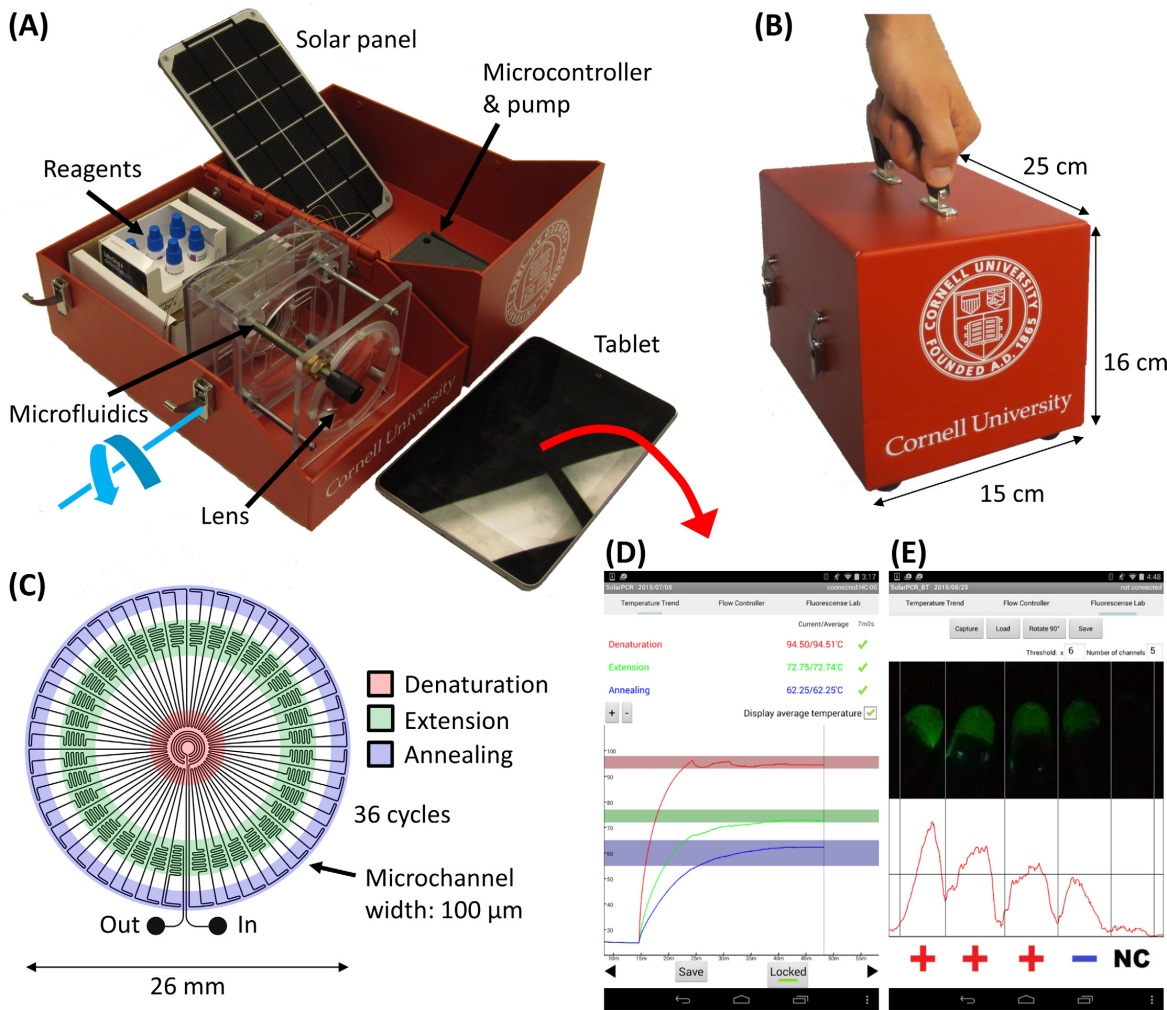
The KS-Detect system. (A) The system contains all components necessary for solar thermal PCR and subsequent analysis, including reagents, tablet, and solar panel. The focusing lens is fixed to the red container on a hinge, allowing rotation (blue arrow) for alignment with the sun. (B) The system is easily carried in one hand, affording easy transportation to patients in remote communities. (C) Microfluidics schematic. Samples are cycled between the warmer center of a PDMS chip (for denaturation of DNA) and the cooler edges (for annealing of primers). A thin, black PDMS layer (not pictured) serves as the bottom of the microfluidic chip, and absorbs solar radiation. (D) Our custom Android application is used to track each temperature zone within the microfluidics and to (E) analyze results via fluorescence levels imaged by a smartphone or tablet.

KS-Detect is a fully contained, portable system capable of both nucleic acid amplification and subsequent analysis (Fig 1A). The major novelty of KS-Detect is that it uses solar thermal energy to heat the sample to temperatures required for the denaturation, extension, and annealing steps of PCR. This allows the system to operate autonomously from traditional energy sources and standard laboratory equipment, making diagnostics available to patients who reside at long distances from clinics, or to patients who receive care at clinics with insufficient diagnostic equipment. The system is organized into a portable kit that allows an operator to easily carry all necessary equipment in one hand (Fig 1B).

The kit contains an Android tablet, solar panel (necessary only when in use for more than 70 hours[19]), microfluidic chip, concentrating lens with alignment fixture, small peristaltic pump, microcontroller, and reagents. The disposable, PDMS microfluidic chip is used to guide a sample through the appropriate thermal profile, achieving continuous flow PCR. The full description of the microfluidic function and design is provided in our previous publication[19]; however, a schematic is shown in Fig 1C and illustrates the basic operating principles. The kit also contains space for biopsy punches, reagents necessary for both HotSHOT DNA extraction and PCR, as well as filters and LEDs used for result analysis. The tablet or smartphone–which may be charged using the solar panel during extended use–provides the power and user-interface to pump the sample through the microfluidic device, to read, display, and store temperature measurements (Fig 1D), and to capture fluorescent images of the amplified samples (Fig 1E). A custom Android app controls all three tasks. Bluetooth support has also been incorporated, allowing wireless control. When using Bluetooth, the microcontroller and pump is powered by a small battery (4.4 Amp-hours), which can also be recharged through the solar panel. KS diagnosis is determined based on fluorescence intensity values extracted from a smartphone image taken after PCR. Integration of a smartphone/tablet for system operation and result analysis allows KS-Detect to be used with minimal training, and helps to decrease system cost. Smartphone ownership and use is growing worldwide–even in resource-limited settings[20]–so it feasible for a healthcare worker to provide his or her own smartphone for use with the KS-Detect.

The procedure for operating the KS-Detect system is as follows. For experiments conducted in the field, a skin biopsy is first obtained from the patient; in this work, we prepared pseudo-biopsy samples from two established human cell lines (see methods for details). Following biopsy, HotSHOT thermal lysis[21] is used to extract DNA from the sample. The extracted DNA is then amplified in a PCR reaction mixture of nuclease-free water, forward/reverse primers, and a dry PCR reagent bead (freeze-dried reagents are pragmatic in resource-limited settings because they are stable for long periods of time at room temperature). The system is manually aligned with the sun and the chip-to-lens distance is adjusted until the temperature zones required for PCR are reached within the microfluidic chip. Modulation of the chip-to-lens distance also gives the system the flexibility to operate in a variety of ambient temperatures. For ambient temperatures ranging from 0°C to 30°C we predict only minor deviations (3°C-6°C) from ideal PCR temperatures[19]. After an appropriate thermal profile is reached, the sample is flowed through the chip, exiting after 36 cycles of solar thermal PCR. Finally, the sample is combined with a fluorescent marker and imaged with a smartphone or tablet. Total time for the procedure is about one hour, with the longest steps being the heating of the chip (about 15 minutes) and the cycling of the sample through the microfluidics (about 30 minutes).

## Results and Discussion

### Pseudo-biopsy characterization and histology

Pseudo-biopsies were used to characterize the performance of the KS-Detect system. Pseudo-biopsies represent controlled samples suitable for engineering performance evaluation, while still mimicking human biopsies as closely as possible. While our previous publication tested PCR performance using plasmid DNA (with a detection limit between 10^5^ and 10^4^ KSHV copies/μL[19]), our pseudo-biopsy samples are made from human cell lines and therefore contain cellular components similar to those found in real biopsies. We prepared pseudo-biopsy samples from a combination of two human B-cell lymphoma cell lines: BC-3 and IBL-1. The BC-3 line contains the KS viral episome, while the IBL-1 line does not[22]. The IBL-1 line is positive for Epstein-Barr virus (EBV), which is the most closely related human virus and used here as a KSHV negative control. We combined BC-3 and IBL-1 cells in varying concentrations (100%, 10%, 1%, and 0% BC-3 cells) and clotted the cells into a solid biopsy-like mass using fibrinogen and thrombin: two common biological proteins that aid in blood clotting. When processed using standard histological techniques, including formalin fixation and paraffin embedding, our pseudo-biopsies appeared similar to human biopsies. Details for pseudo-biopsy preparation can be found in the methods section of this paper.

**Fig 2:**
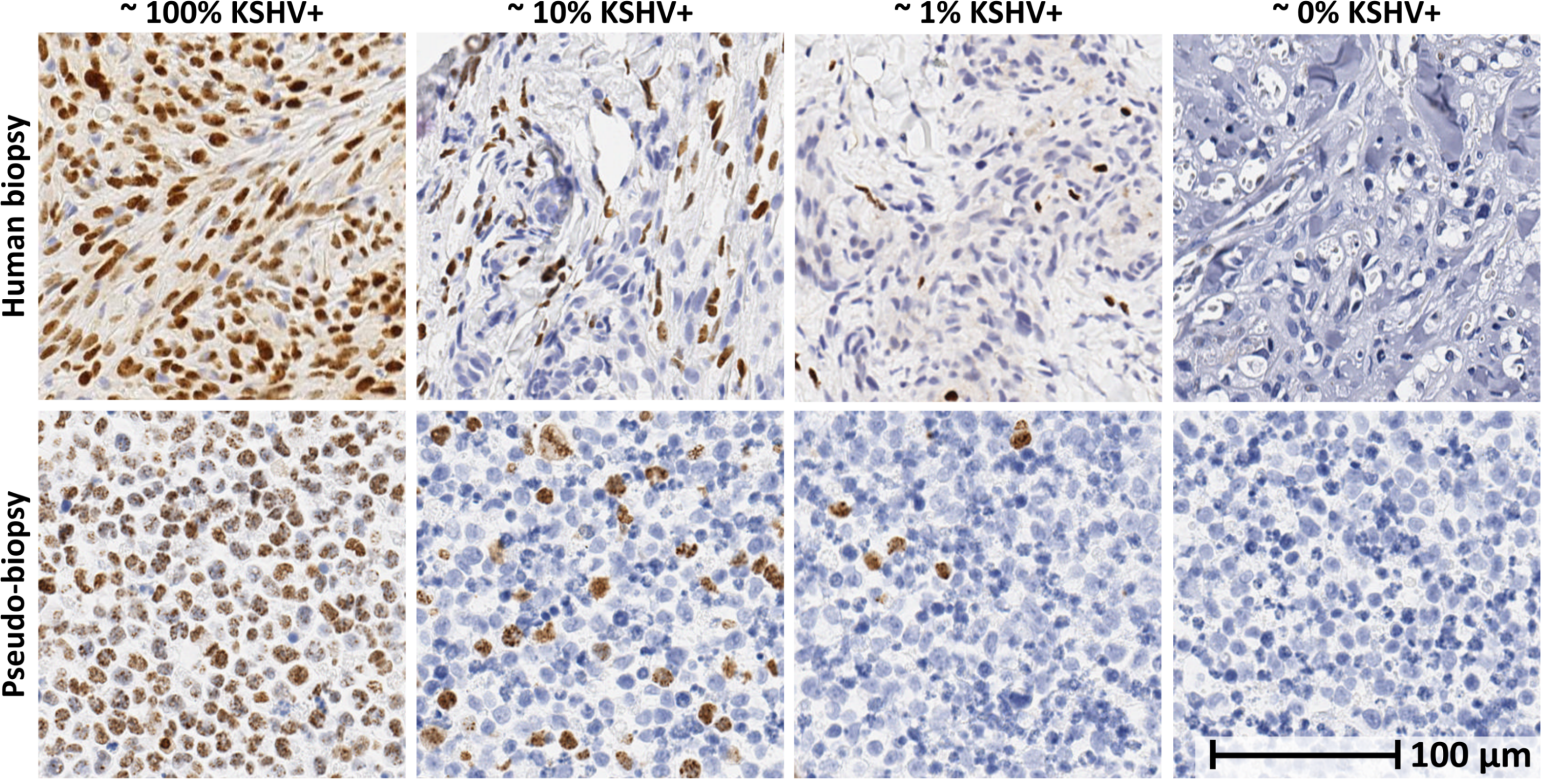
Visual comparison of pseudo-biopsy and human biopsy samples. Cell block pseudo-biopsies with varying KS-positive cells (bottom) were used to imitate human biopsies (top) to validate function of the device. Cell block pseudo-biopsies were embedded in paraffin and stained for latency-associated nuclear antigen (LANA) for comparison with LANA stained human biopsies. KSHV-infected nuclei are brown with dark punctae, while uninfected nuclei are blue. Images of human biopsies were taken from representative sections of KS-positive samples with concentrations similar to the cell block biopsies by visual inspection. The 0% image was taken from an uninfected region of a human sample with low concentration of KS positive cells. Precise analysis of infected cell percentage per sample was performed using HALO image analysis software (Indica Labs), and can be found in S1–S5 Figs. Scale bar applies to all images.

To objectively assess the number of infected cells in our pseudo-biopsies, we used image analysis after performing immunohistochemistry for the KSHV protein LANA. The image analysis algorithm corroborated that close to 100%, 10%, 1%, and 0% of the cells were positive for KSHV (Fig 2 and S1–S5 Figs). We used the HotSHOT extraction technique to obtain DNA from the pseudo-biopsies, which requires two simple and inexpensive solutions that can be stored at room temperature, and a 30 minute boiling step, but no specialized laboratory equipment[21]. Using this extraction method, we verified using conventional PCR and gel electrophoresis the presence of DNA and sensitivity of detection to the 1% level. These same DNA samples were then used to test the KS-Detect System.

### KS-Detect results: gel electrophoresis

**Fig 3:**
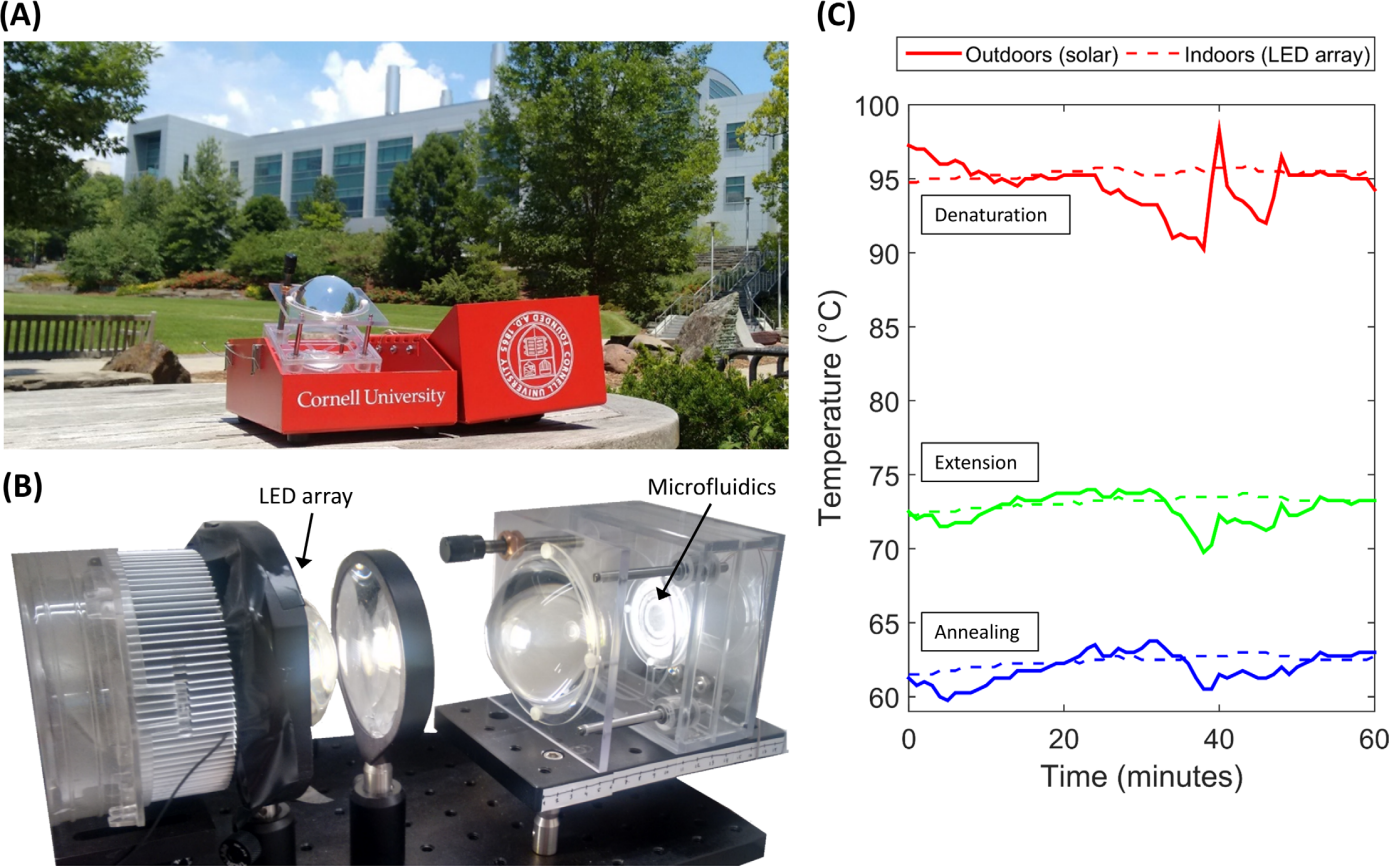
Light source comparison: indoors and outdoors. (A) The KS-Detect system during an experiment using sunlight. Lens must be manually aligned with the sun as the sun moves across the sky. (B) For experiments conducted indoors, the lens and microfluidics are removed from our portable kit, and are fixed in front of a 100 Watt LED array. (C) A typical solar temperature profile is more variable when compared to a typical LED array temperature profile, due to cloud coverage (as seen at about 35 min.) and intermittent realignment of the lens with the sun.

DNA amplification was conducted using both sunlight and a LED array as heat sources. The LED array allows us to test our system indoors using constant thermal boundary conditions, giving a result that might resemble those obtained in ideal weather conditions. Fig 3A shows the KS-Detect outdoors during an experiment using sunlight, and Fig 3B shows our indoors setup which uses a 100 Watt LED array. While the observed temperature profile when using sunlight for heating was sufficiently stable for PCR, we observed a more-consistent temperature profile when using the LED array for heating. Fig 3C shows an example of typical outdoors and indoors temperature profiles over an hour.

**Fig 4:**
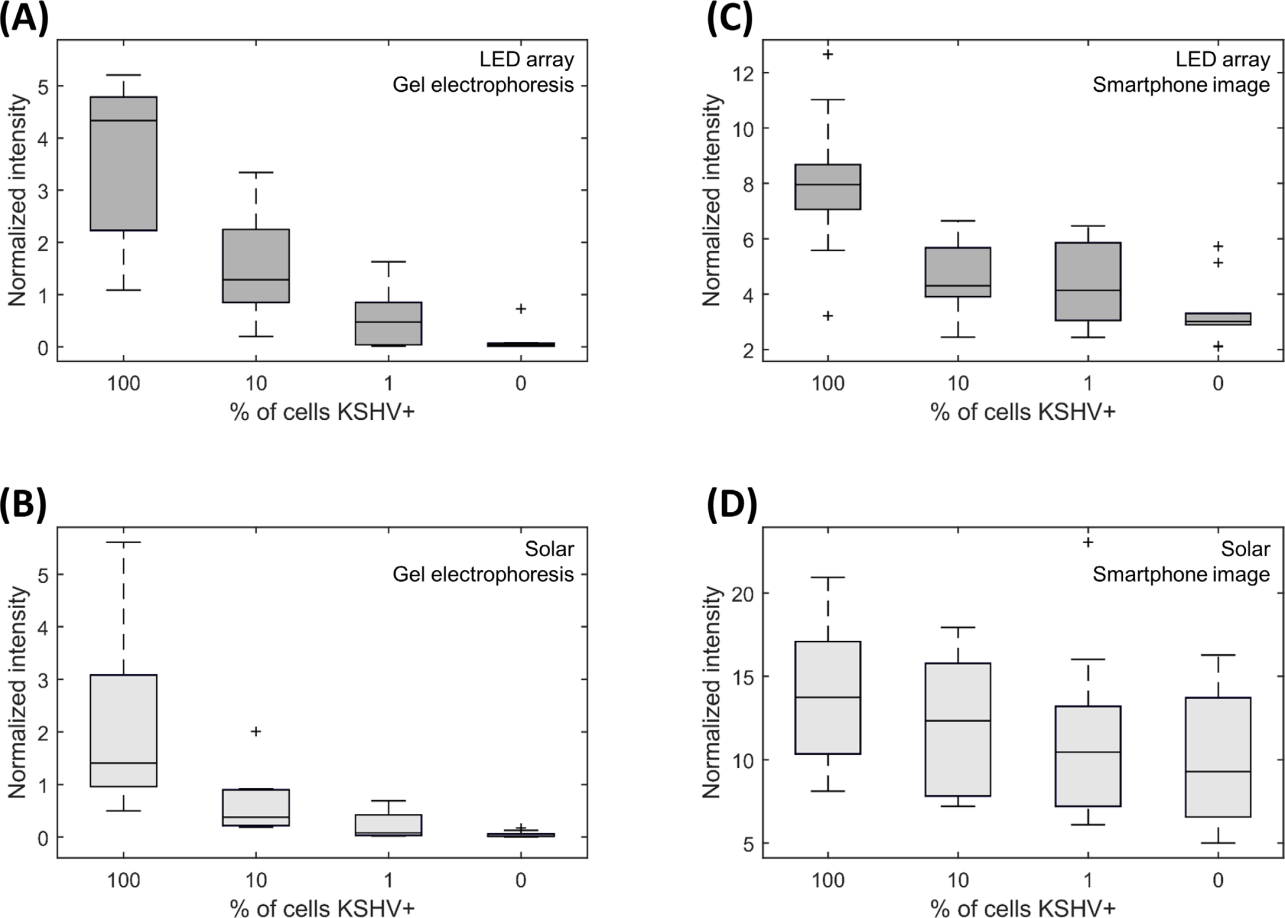
Pseudo-biopsy amplification results. (A, B) Results when analyzed by standard gel electrophoresis. (C, D) Results when analyzed by smartphone image (as seen in Fig 1E). 40 trials were completed with each of the two heating methods, with 10 trials at each of four KSHV+ concentrations (overall number of trials: 80). Data represented by darker boxes (A, C) were heated indoors by LED array, while data represented by lighter boxes (B, D) were heated outdoors via sunlight. Box plots were made with standard conventions: boxes range from the 25th to 75th percentiles, and whiskers extend to a maximum of 1.5x the interquartile range.

We amplified samples of varying KSHV+ cell concentrations using both heat sources, and first quantified the results using standard gel electrophoresis. Our primers were designed to amplify a 164 bp subfragment of the KSHV episome, in the viral cyclin (vcyclin) gene (ORF72). Fig 4A shows 164 bp intensity values obtained through gel electrophoresis when the KS-Detect was operated indoors via LED array. Both 100% KSHV+ and 10% KSHV+ samples produced signals quite distinct from the negative control (0% KSHV), while sensitivity at 1% KSHV+ diminished. When the KS-Detect was operated outdoors in sunlight (Fig 4B) a similar trend resulted, but 164bp intensity values dropped overall. We therefore hypothesize that uneven heating conditions lead to less-efficient amplification. Uneven heating conditions may arise from clouds as shown in Fig 3C, or from the chip, lens, and sun not being perfectly aligned for the entire experiment (a result of intermittent, manual realignment while the sun moves across the sky).

### KS-Detect results: smartphone images using SYBR green dye

In the field, fluorescent measurements using SYBR green dye provide an easy, portable method for detecting PCR amplicons. SYBR green dye binds to all dsDNA, producing a fluorescent intensity proportional to the amount of DNA present in solution. We mixed SYBR green dye with our amplified samples, illuminated with a blue light, and imaged the fluorescence on a standard Android smartphone through a dichroic filter. Fig 4C shows the results for samples amplified indoors by LED array, and Fig 4D shows the results for samples amplified outdoors by sunlight. Overall, fluorescence difference between high and low KSHV+ samples was diminished when measured by smartphone image as compared to gel electrophoresis. It is interesting to note that samples heated by the LED array still showed a distinct fluorescence difference between high and low KSHV+ samples when measured by smartphone image, while samples heated by the sun were not as distinguishable from one another. Additionally, overall normalized intensity values were about twice as high for solar-amplified samples versus LED array-amplified samples, suggesting a higher presence of non-specific amplicons in the solar-amplified samples. While measuring PCR product via smartphone is an easy and realistic solution for disease diagnosis in the field, concerns do exist. Our results show that if heating conditions cannot be made optimal (such as in Fig 4C), non-specific amplification may prove to overwhelm the signal produced by the target amplicon.

### Sensitivity and specificity of KS-Detect

Using both the gel electrophoresis and smartphone image measurements, we compiled sensitivity and specificity values for the KS-Detect (Table 1). Criteria for determining positive trials can be found in the methods section. Gel electrophoresis proved to be the most sensitive method, as it was possible to distinguish the target amplicon band from non-specific PCR products. Additionally, we can see that KS-Detect achieved higher specificity when considering samples amplified indoors (by the LED array) versus those amplified outdoors (by sunlight). We believe that the sensitivity and specificity of the system could be improved to the indoors level if a more robust solution was implemented to align the lens and microfluidic chip with the sun, as this might reduce non-specific amplification. Overall system sensitivity and specificity was determined to be 83% and 70% respectively, considering the average of the indoors and outdoors trials for the smartphone image results. Furthermore, since we performed these measurements on samples derived from human lymphoma cells using a simple DNA extraction method (HotSHOT), we expect a similar level of sensitivity and specificity on human biopsy samples collected in the field with ≥10% KSHV+ tumor cells.

**Table 1:** Sensitivity and specificity of the KS-Detect system.

Diagnostic method	Gel electrophoresis		Smartphone image	
Heating method	LED array	Solar	LED array	Solar
Sensitivity: high concentration*	100%	90%	90%	75%
**Specificity**	90%	100%	80%	60%

Overall number of trials: 80. 40 tests were conducted indoors (10 per concentration) and 40 were conducted outdoors.

*100% and 10% KSHV+ samples were considered true positives (1% KSHV+ was not included).

### Non-specific amplification in pseudo-biopsy samples

**Fig 5:**
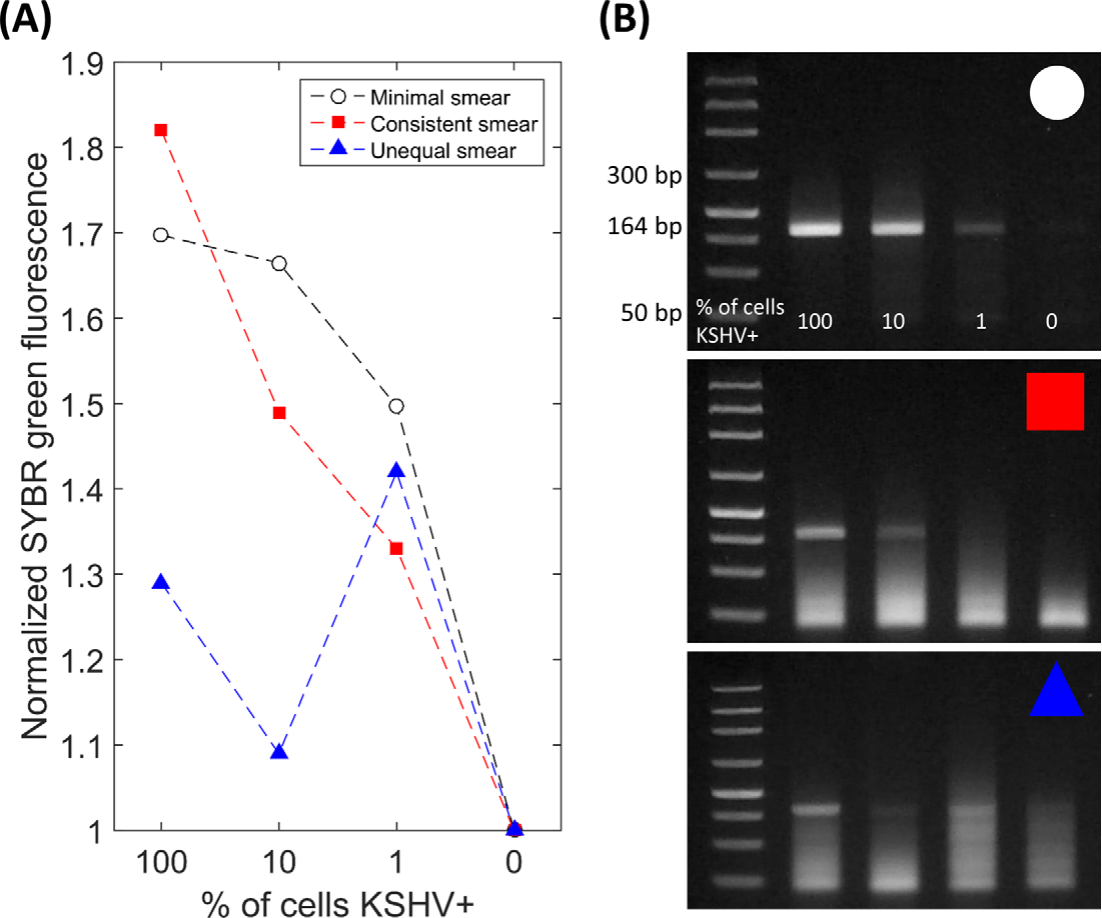
Effect of non-specific amplification on SYBR analysis. (A) SYBR green dye fluorescent intensities with (B) corresponding gel electrophoresis images. Amplification was performed by the KS-Detect system on pseudo-biopsy samples. Some amplification sets (B, top) showed minimal amounts of non-specific amplification (or smearing), while other sets had consistent (B, middle) or variable (B, bottom) amounts across different KSHV+ cell concentrations. For all three gel electrophoresis images, the order of the samples in each well is: DNA ladder, 100%, 10%, 1%, and 0% KSHV+ cell concentrations.

Variable amounts of non-specific amplification were observed in our experiments using pseudo-biopsy samples. It was often strongest around the 50 bp region, which may suggest primer-dimer formation. We performed an analysis of how non-specific amplification (or smearing) affected smartphone image fluorescent levels (Fig 5A). We compared scenarios when there was little to no non-specific amplification (Fig 5B, top), consistent non-specific amplification (Fig 5B, middle), or inconsistent non-specific amplification (Fig 5B, bottom). As expected, our results show that SYBR green dye was only effective when non-specific amplification was minimal or when it was consistent across different KSHV+ concentrations. We conclude that SYBR green can only be recommended for use in the KS-Detect if non-specific amplification is minimal (achievable if there are optimal heating conditions, such as shown in Fig 4C); otherwise, a specific fluorescent marker should be implemented.

### Future system improvements

Technical improvements could be incorporated into the KS-Detect that would further improve its usability. Our comparison of light sources showed that an improvement in amplification efficiency can be achieved if thermal boundary conditions are kept constant during PCR. Currently, manual re-alignment between the sun and the focusing lens is needed intermittently, causing dips and peaks in temperatures as the lens focuses sunlight off-center of our microfluidic chip. An automated system could be built so that the focusing lens is always aligned with the sun. Such automation would come at the cost of increased energy requirements, but should increase amplification efficiency, and would make the device easier to operate. Additionally, non-ideal weather (i.e. cloud coverage) drop temperatures from optimal values. Future systems should implement designs with high heat capacitance, allowing temperatures to remain stable during brief times of cloud coverage.

Specific fluorescent markers could be used into the final smartphone image analysis, and would negate the effects of non-specific amplification. Previous work using plasmid DNA samples showed little non-specific amplification[19], suggesting that PCR inhibitors (perhaps resulting from the HotSHOT extraction method) interfered with the primers’ affinity to bind to their targets, or gave rise to non-specific hybridization to cellular or EBV sequences. We attempted to optimize primer design (S6 Fig), and our results (S7 Fig) show that non-specific amplification may be reduced further by primer optimization. The use of fluorescent hydrolysis probes such as molecular beacons may provide a means to bypass fluorescence contribution due to non-specific amplicons, but our attempts with this approach have failed thus far. We hypothesize that this may be due to a lack of available ssDNA available for hydrolysis with molecular beacons. In future endeavors, it may be possible to use asymmetric PCR to intentionally amplify an excess of ssDNA[23], which could allow for the use of molecular beacons as end-point PCR fluorescent markers. Alternatively, KS amplicons could be detected in the field using gold and silver nanoparticles[24].

## Conclusions

We have demonstrated the utility of the KS-Detect system and solar thermal PCR for the identification of KSHV using samples similar to human biopsies. We achieved 83% sensitivity and 70% specificity when diagnosing by smartphone image using ≥10% KSHV+ pseudo-biopsy samples, which visually matches the concentration of LANA stained KSHV+ cells from histology found in other published works[1,25–27]. The KS-Detect system contains all equipment necessary for DNA extraction from skin biopsies, solar thermal PCR, and result analysis. The KS-Detect can be operated with a user’s smartphone or tablet, and fulfills energy requirements with sunlight instead of electricity, enabling diagnosis in remote areas. We have identified that a uniform solar heating profile is important for efficient and specific amplification of target, and that results from non-specific fluorescent markers (such as a SYBR green dye) in particular can benefit from consistent, reliable solar heating profiles. Future solar thermal PCR devices might incorporate autonomous solar-tracking designs to achieve more efficient PCR and should consider methods to enable efficient operation in non-ideal weather conditions (e.g. systems with high heat capacitance). Alternatively, if engineering restraints prevent the previous design recommendations, specific hydrolysis probes should be used for result analysis to negate the adverse effects of non-specific amplification.

We have motivated our system with Kaposi’s sarcoma in mind, but primer modification would make our device useful for the point-of-care diagnosis of other diseases common in locations with unique energy needs or portability requirements. Some studies suggest that decentralized care is superior to centralized hospitalization in resource-limited settings. In the case of tuberculosis–a disease in which molecular diagnostics is also effective–time to treatment has shown to be significantly reduced through the use of a decentralized care approach[16,28,29]. Owing to its portability and energy flexibility, the KS-Detect system could be a useful tool for such applications. Our results shown here–using samples closely resembling human biopsies–validate the KS-Detect as a viable, portable molecular diagnostic tool.

## Materials and Methods

### Pseudo-biopsy preparation

Pseudo-biopsy samples were prepared from established human cell lines: BC-3 and IBL-1[22,30]. After expansion of the BC-3 (KSHV+) and IBL-1 (EBV+, KSHV-) cells in culture, the number of cells within a flask was determined using an optical cell counter. Suspensions of BC-3 and IBL-1 cells were aliquoted and combined such that each new suspension contained 20 million cells total at differing ratios of BC-3 to IBL-1 cells. The combined suspensions were well mixed and then pelleted at 400 g for 5 minutes. The supernatant was discarded and the pellets were transferred to microcentrifuge tubes. The pellets were resuspended in 50 μL of 5 mg/mL fibrinogen (Sigma-Aldrich, cat. no. F3879-1G, in 90% RPMI 1640, 5% 1X phosphate buffered saline, and 5% ultrafiltered water) and 5 μl of 1 U/μl Thrombin (Biovision, cat. no. 7591–1 in 0.9% NaCl) was added to the suspension. When mixed together, thrombin catalyzes the polymerization of fibrinogen into fibrin. In our setup, the cells became locked in place as the fibrinogen crosslinked around each cell. The clotted cells were centrifuged at 8000 rpm for 15 seconds to further pack the cells together. The completed pseudo-biopsies have similar textural consistency to human skin lesion biopsies and reveal microscopic similarity when processed using standard histological techniques.

### HotSHOT DNA extraction

After pseudo-biopsy preparation, DNA extraction was conducted using the HotSHOT procedure. HotSHOT extracts DNA through heating of the samples in an alkaline lysis reagent at 95°C[21]–a task that our system can achieve through solar thermal heating. An alkaline lysis reagent was prepared by adding 25 mM NaOH and 0.2 mM disodium EDTA in water to a pH of 12. A neutralizing reagent with 40 mM Tris-HCl was prepared by dissolving salts in water to a pH of 5. Biopsy samples were collected and placed in a tube with 75 μL alkaline lysis reagent and heated to 95°C for 30 minutes. Samples were then cooled to 4°C and 75 μL of the neutralizing reagent was added.

### Solar thermal PCR reaction mixture

We combined one PuReTaq™ Ready-To-Go™ PCR bead (GE Healthcare), 25 μL of 0.4% w/v polyvinylpyrrolidone in DI water, 0.5 μL of 5 Units/μL Taq polymerase (AmpliTaq®, Life Technologies), 0.5 μL each of our forward and reverse primers (starting concentration 50 μM), and 1 μL of HotSHOT extracted DNA from a pseudo-biopsy sample. PCR beads from GE Healthcare are dry beads containing all necessary reagents for PCR except for water, primers, and template DNA, and can be stored at room temperature. Although PCR beads from GE Healthcare contain 2.5 units of Taq polymerase, we observed improved amplification results when adding extra Taq polymerase (about 2.5 Units), suggesting adsorption of Taq polymerase into our PDMS microfluidics, despite BSA pre-treatment. This observation is supported by the fact that extra Taq polymerase was not necessary for PCR conducted in a standard thermal cycler (see supporting information).

### Solar thermal PCR

PDMS microfluidic chips were treated with bovine serum albumin (BSA) overnight in a humidity chamber at 4°C. BSA helps to prevent adhesion of reagents onto the surfaces of the PDMS microchannels[31]. After 24 hours, the chips were flushed clear of the BSA with water, then with air. When running an experiment, a new microfluidic chip was inserted into the alignment fixture. The chip was heated to a PCR-appropriate temperature profile by pointing the lens towards the sun. It was possible to fine-tune the temperatures by changing the distance between the lens and the chip. While the chip was heating, about 60 μL of paraffin oil was pumped through the chip at 5 μL/min. We observed significant absorption of paraffin oil into the PDMS microchannels. Once the correct temperatures were reached, about 15 μL of our target sample was sent through the chip at an approximate rate of 1 μL/min. The sample was followed by another oil plug, preventing expansion and evaporation of the aqueous sample. After amplification, we deposited the sample into a small thermal cycler tube for later analysis. For experiments conducted with our LED array, the same procedure was used, except lens alignment and the lens-to-chip distance was preset and fixed.

### Gel electrophoresis

The Lonza Flashgel™ system was used for our gel electrophoresis measurements. 10 μL of water and 2 μL of amplified sample (or DNA ladder 50 bp—1.5 kb, Lonza cat. no. 57033) was added to each well in the premade gels (Lonza cat. no. 57031). An electric potential of 270 volts was applied to the gel for 6 minutes, and images were recorded under UV light using a digital camera. Images were inspected with ImageJ’s Gel Analyzer tool using standard procedure. Intensity values were normalized by the 150 bp marker of the DNA ladder in each image, and the 164 bp target band was deemed positive if its intensity was at least one-fifth of the 150 bp marker. The 150 bp band was chosen as a reference because it was closest in size to the 164 bp target amplicon. Sensitivity was taken as the ratio of the number of positive results to the number of amplifications performed, considering only the 100% and 10% KSHV+ samples. Specificity was taken as the ratio of the number of negative results to the number of amplifications performed, considering only the 0% KSHV+ concentration sample.

### Smartphone based fluorescence detection

5 μL of SYBR green DNA dye (Life Technologies 10,000X concentrate diluted to 10X in TBE buffer) was combined with 1 μL of amplified sample. A 465 nm blue LED was used as an excitation source, and the sample was viewed through a dichroic filter (505–575 nm). An image was taken with a smartphone and analyzed through both ImageJ and our in-house Android application. Mean intensity values were then extracted using ImageJ. For each solar thermal PCR trial, a control sample of SYBR green and DI water was viewed simultaneously with the amplified samples. The intensities of the amplified samples were then normalized by the water control. A sample was taken as positive if its mean fluorescence was greater than the mean fluorescence of all the 0% KSHV+ samples for its particular heating method (either LED array or solar). Sensitivity was taken as the ratio of the number of positive results to the number of amplifications performed, considering only the 100% and 10% KSHV+ samples. Specificity was taken as the ratio of the number of negative results to the number of amplifications performed, considering only the 0% KSHV+ concentration sample.

## Supporting Information

S1 Dataset Gel electrophoresis and smartphone image raw intensity values. (XLSX)

S1 Fig Positive cell counts in human biopsy and pseudo-biopsy samples. To confirm KSHV+ cell concentrations, HALO image analysis software from Indica Labs was used to count the number of positive cells in both pseudo-biopsy and human biopsy samples. Total nuclei and percentage of nuclei positive for KSHV are given.
(TIF)

S2 Fig Pseudo-biopsy histology slide (100% KSHV+). (**Figure A**) Histology image. (**Figure B**) Histology image overlaid with masking algorithm from HALO image analysis software (green shows positive nuclei, blue shows negative nuclei). Image taken at 20X magnification. This block is known to contain 100% BC-3 cells (KSHV+) but the masked image shows multiple blue nuclei and the software calculated the percent infection to be 87%. The error in this calculation can be attributed to the limits of the image analysis algorithm as well as the limits of the LANA stain.
(TIF)

S3 Fig Pseudo-biopsy histology slide (10% KSHV+). (**Figure A**) Histology image. (**Figure B**) Histology image overlaid with masking algorithm from HALO image analysis software (green shows positive nuclei, blue shows negative nuclei). Image taken at 20X magnification.
(TIF)

S4 Fig Pseudo-biopsy histology slide (1% KSHV+). (**Figure A**) Histology image. (**Figure B**) Histology image overlaid with masking algorithm from HALO image analysis software (green shows positive nuclei, blue shows negative nuclei). Image taken at 20X magnification.
(TIF)

S5 Fig Pseudo-biopsy histology slide (0% KSHV+). (**Figure A**) Histology image. (**Figure B**) Histology image overlaid with masking algorithm from HALO image analysis software (green shows positive nuclei, blue shows negative nuclei). Image taken at 20X magnification.
(TIF)

S6 Fig Comparison of PCR primer pairs. Three primer pairs were tested, all producing 164 bp amplicons. Primer pair B produced the least amount of non-specific amplification when amplifying KS pseudo-biopsy samples. Primer sequences are reported 5’ to 3’.
(TIF)

S7 Fig Standard PCR results using different primer pairs. Gel electrophoresis image comparing non-specific amplification when amplifying psuedo-biopsies in a standard thermal cycler, using a variety of DNA primers. All primer pairs were cross-referenced with the EBV episome, with no matches found.
(TIF)

S8 Fig Comparison of solar thermal PCR to conventional PCR when amplifying pseudo-biopsy samples. Our results show a decrease in amplification efficiency when amplifying by our solar thermal PCR system, as compared to a standard thermal cycler. However, 164 bp bands are still visible for pseudo-biopsies prepared with 100%, 10% and 1% KSHV+ cells.
(TIF)
