# Supplementary figures and images for: KS-Detect – Validation of Solar Thermal PCR for the Diagnosis of Kaposi’s Sarcoma Using Pseudo-Biopsy Samples
Source: PLoS One. 2016 Jan 22;11(1):e0147636. doi: 10.1371/journal.pone.0147636 (PMC4723253; doi:10.1371/journal.pone.0147636)

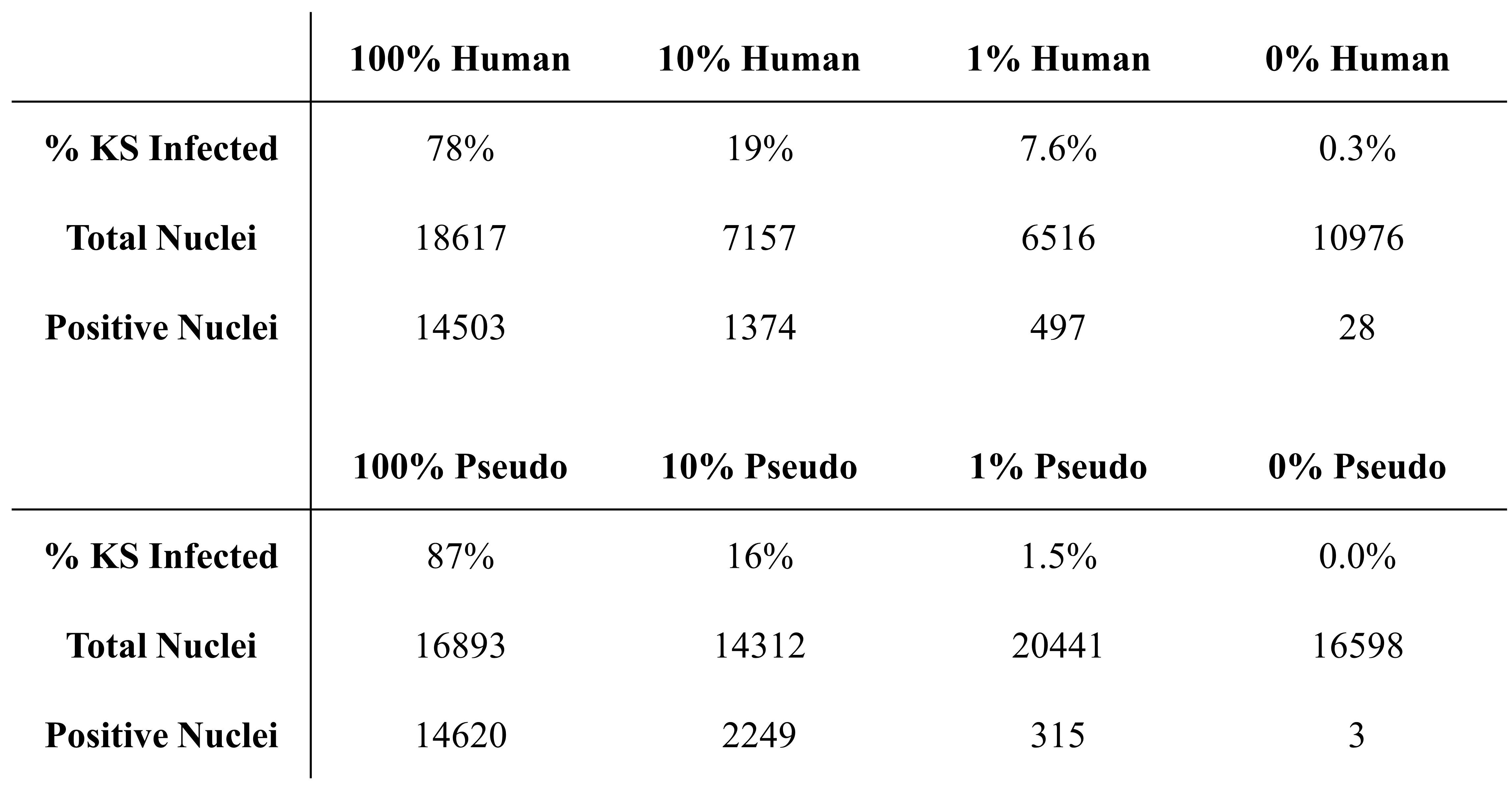

Supplement: S1 Fig — To confirm KSHV+ cell concentrations, HALO image analysis software from Indica Labs was used to count the number of positive cells in both pseudo-biopsy and human biopsy samples. Total nuclei and percentage of nuclei positive for KSHV are given. (TIF) [file pone.0147636.s002.tif]

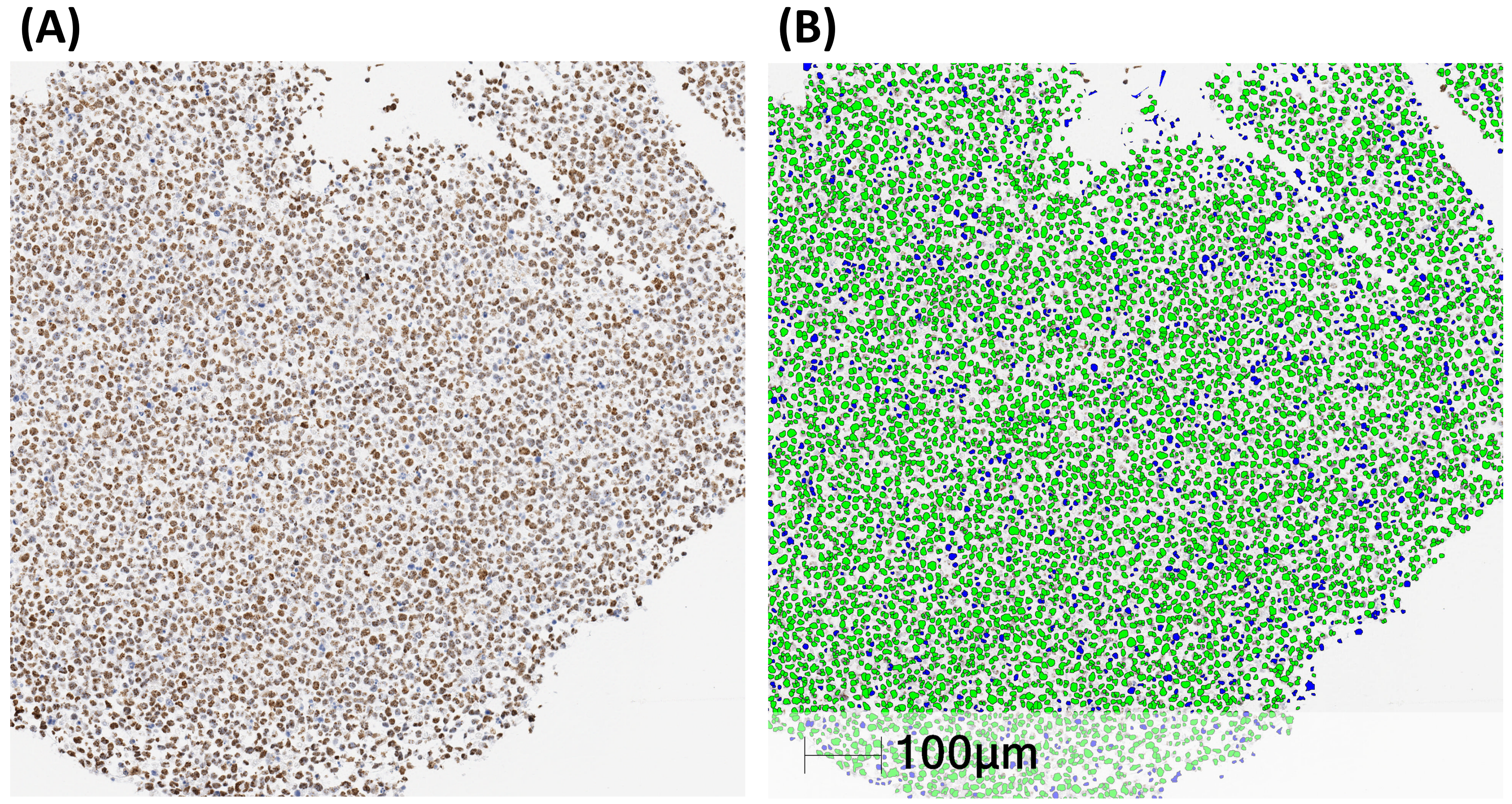

Supplement: S2 Fig — (Figure A) Histology image. (Figure B) Histology image overlaid with masking algorithm from HALO image analysis software (green shows positive nuclei, blue shows negative nuclei). Image taken at 20X magnification. This block is known to contain 100% BC-3 cells (KSHV+) but the masked image shows multiple blue nuclei and the software calculated the percent infection to be 87%. The error in this calculation can be attributed to the limits of the image analysis algorithm as well as the limits of the LANA stain. (TIF) [file pone.0147636.s003.tif]

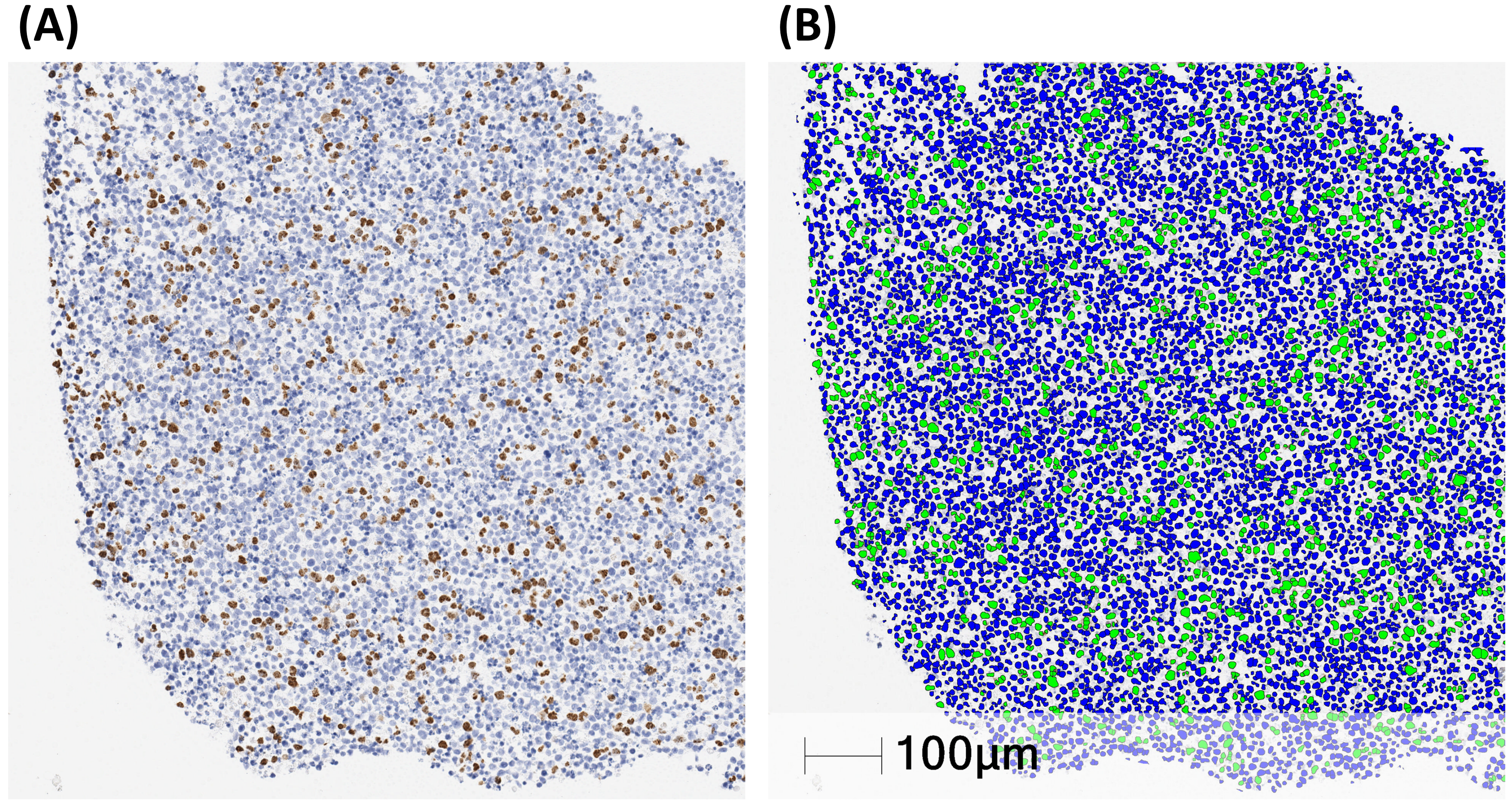

Supplement: S3 Fig — (Figure A) Histology image. (Figure B) Histology image overlaid with masking algorithm from HALO image analysis software (green shows positive nuclei, blue shows negative nuclei). Image taken at 20X magnification. (TIF) [file pone.0147636.s004.tif]

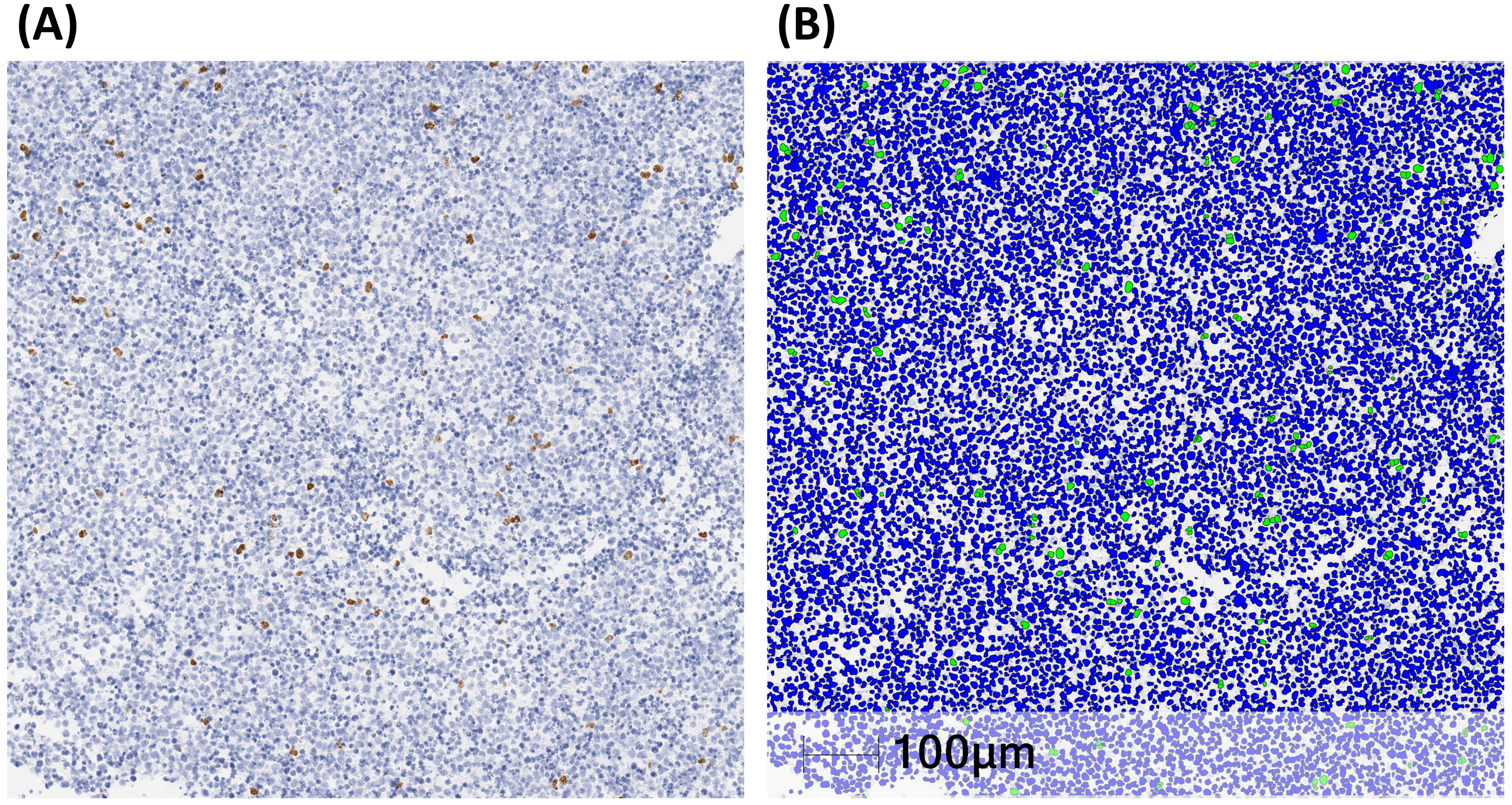

Supplement: S4 Fig — (Figure A) Histology image. (Figure B) Histology image overlaid with masking algorithm from HALO image analysis software (green shows positive nuclei, blue shows negative nuclei). Image taken at 20X magnification. (TIF) [file pone.0147636.s005.tif]

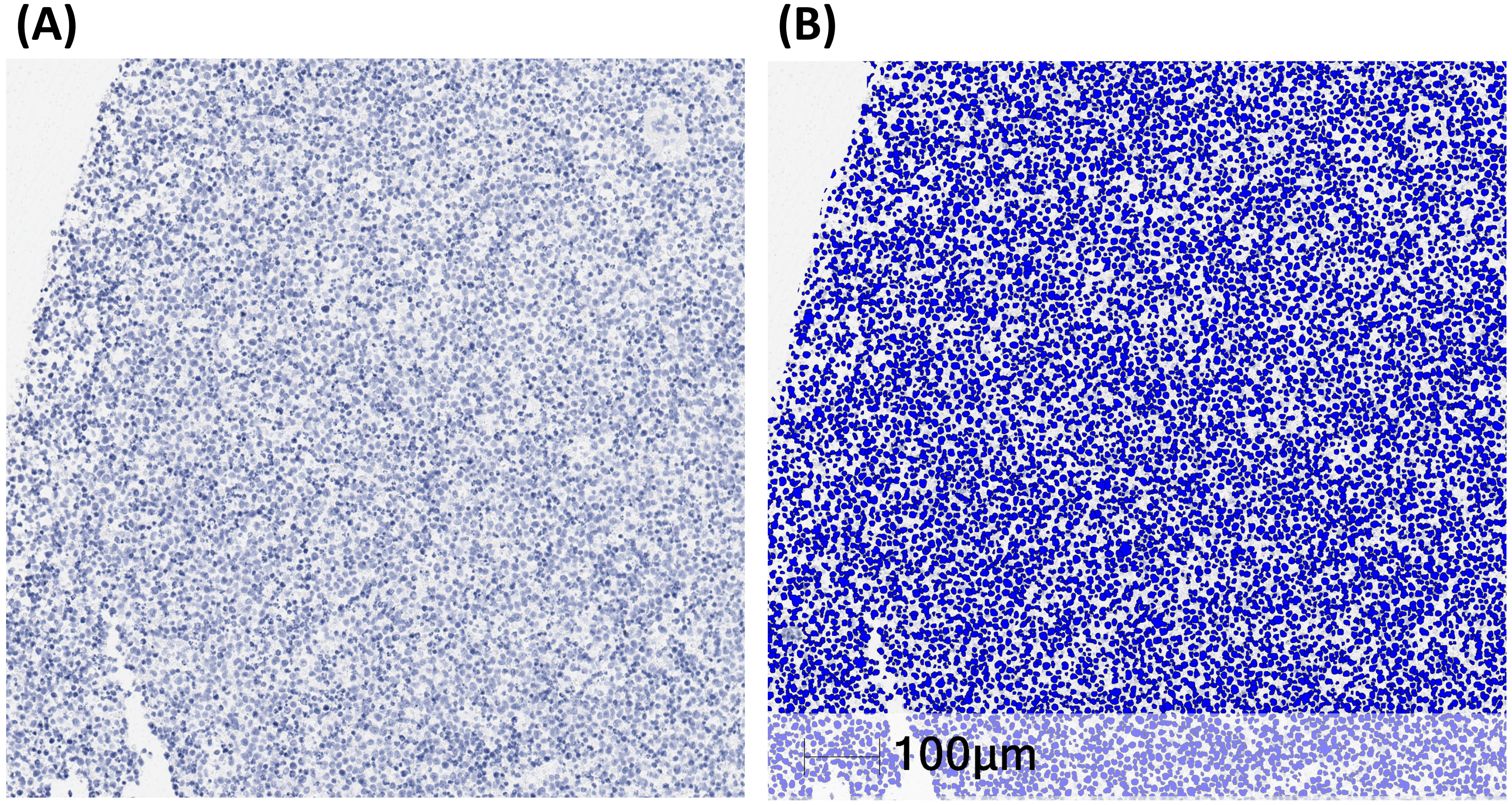

Supplement: S5 Fig — (Figure A) Histology image. (Figure B) Histology image overlaid with masking algorithm from HALO image analysis software (green shows positive nuclei, blue shows negative nuclei). Image taken at 20X magnification. (TIF) [file pone.0147636.s006.tif]

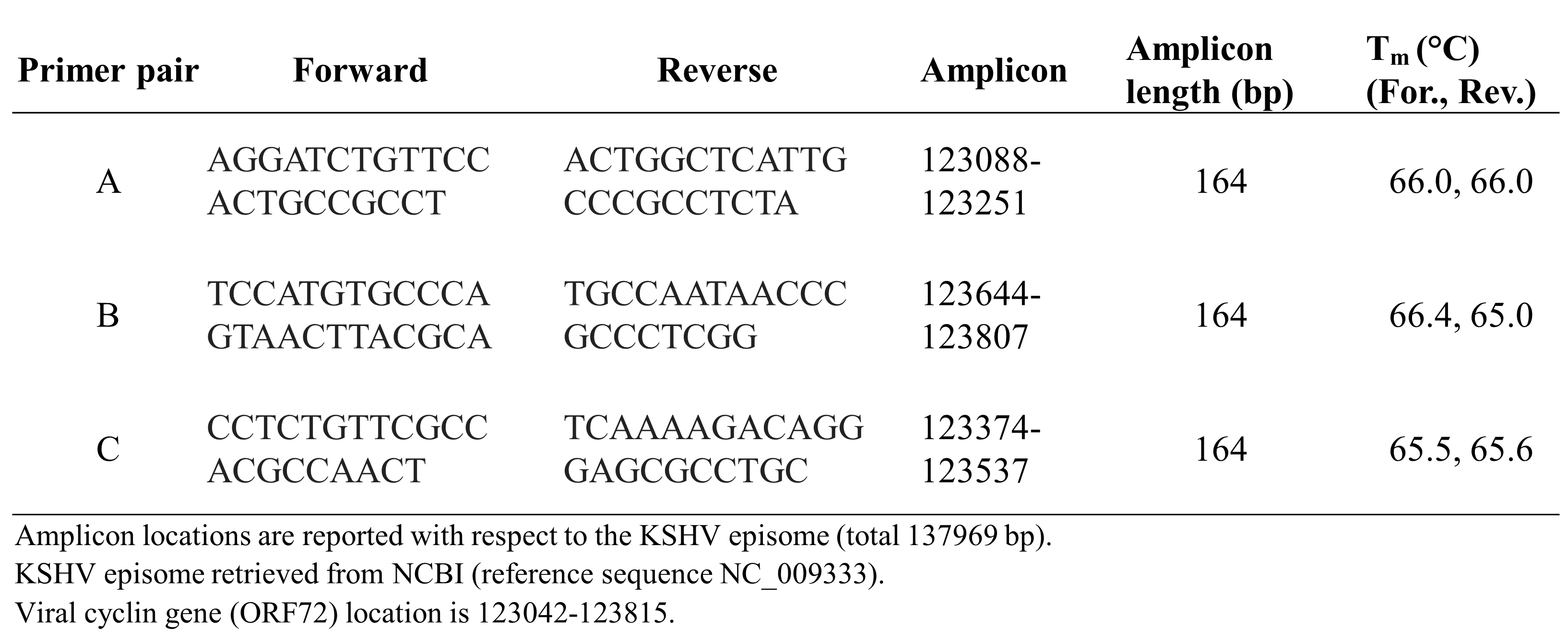

Supplement: S6 Fig — Three primer pairs were tested, all producing 164 bp amplicons. Primer pair B produced the least amount of non-specific amplification when amplifying KS pseudo-biopsy samples. Primer sequences are reported 5’ to 3’. (TIF) [file pone.0147636.s007.tif]

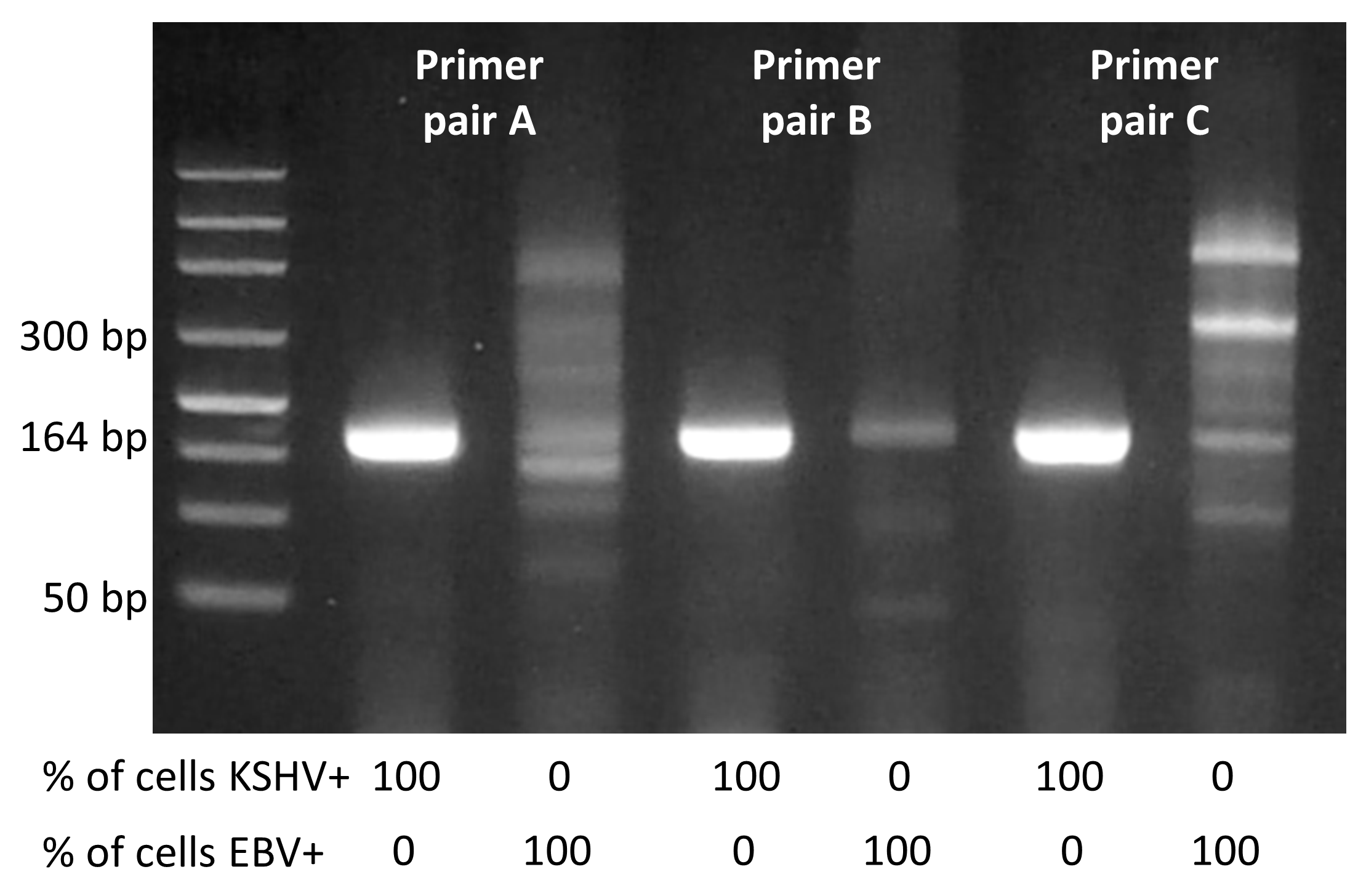

Supplement: S7 Fig — Gel electrophoresis image comparing non-specific amplification when amplifying psuedo-biopsies in a standard thermal cycler, using a variety of DNA primers. All primer pairs were cross-referenced with the EBV episome, with no matches found. (TIF) [file pone.0147636.s008.tif]

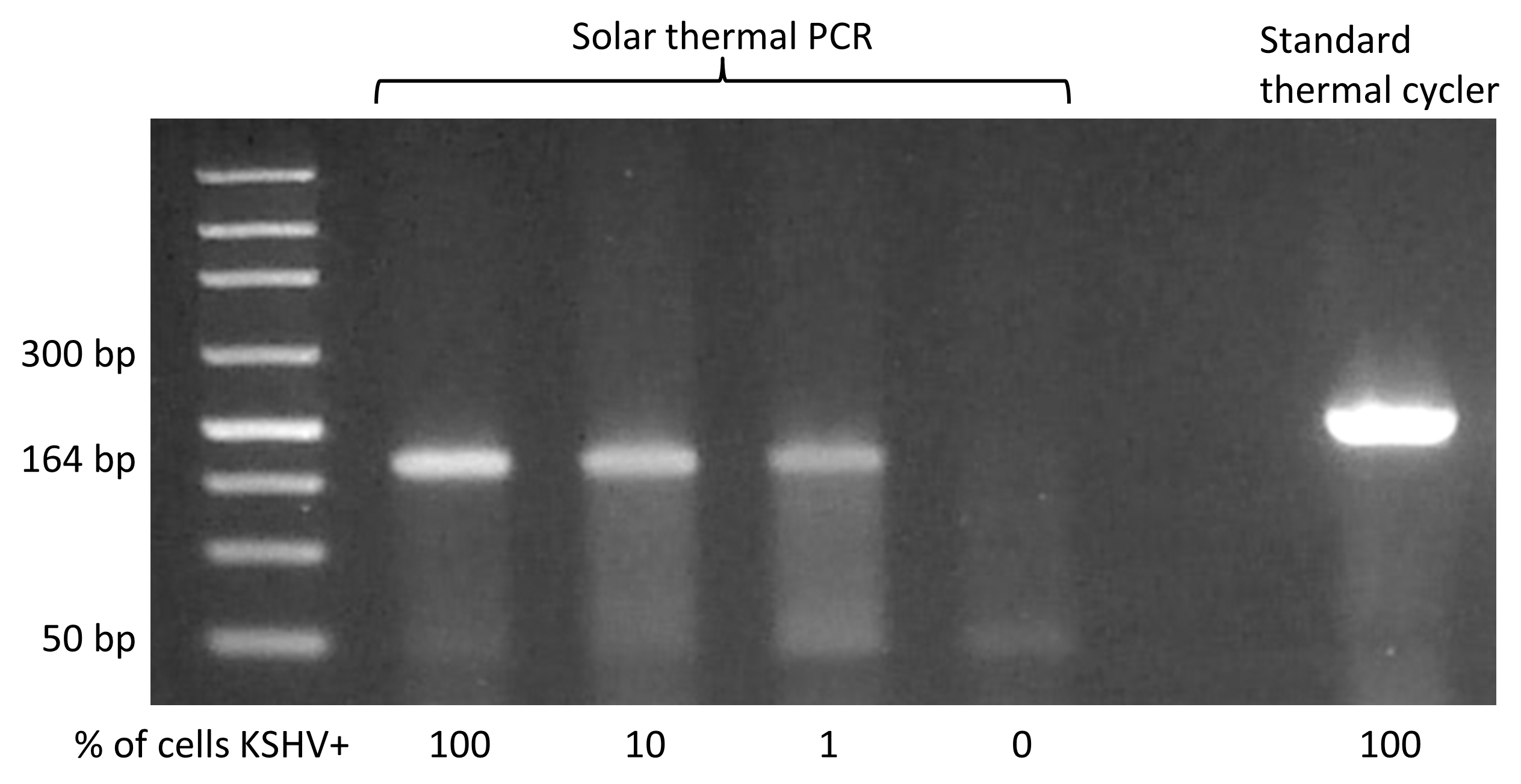

Supplement: S8 Fig — Our results show a decrease in amplification efficiency when amplifying by our solar thermal PCR system, as compared to a standard thermal cycler. However, 164 bp bands are still visible for pseudo-biopsies prepared with 100%, 10% and 1% KSHV+ cells. (TIF) [file pone.0147636.s009.tif]
